# Case of Rupture of Middle Meningeal Artery: Operation; Recovery

**Published:** 1908-03

**Authors:** G. Munro Smith

**Affiliations:** Surgeon to the Bristol Royal Infirmary


### CASE OF RUPTURE OF MIDDLE MENINGEAL ARTERY: OPERATION; RECOVERY.

BY

G. MUNRO SMITH,

*Surgeon to the Bristol Royal Infirmary.*

---

THE following case is, I think, worth a brief record, because of its intrinsic interest, and because the symptoms and physical signs were throughout typical of the lesion, and led to early and successful treatment.

A. L., aged four years, fell out of a cart on June 14th, 1907, and was taken up in a stunned condition. He soon regained his senses and seemed fairly well until the evening of the following day, when he became rather suddenly unconscious, and was at once brought from his home at Hambrook to the Bristol Royal Infirmary.

On admission he was unconscious, but occasionally cried out and moved his limbs restlessly about. It was noticed that he moved his right side much more freely than his left. His pulse was 60, his respirations quiet, with a long-drawn breath now and then, almost like a yawn. The temperature in the right axilla was 99° F., and in the left 98°.

The pupils were equal in size, and reacted to light. Soon after admission, however, the right (side of lesion) became dilated and fixed.

On the right temporal region of the head was an oval, flat, puffy swelling some two or three inches in diameter. No depression or fracture could be felt.

The clinical history, the site of the swelling, the coma, the dilatation of the right pupil, and the partial loss of voluntary movement on the opposite side to the injury, &c., immediately suggested rupture of the middle meningeal artery, and it was decided at once to open the skull.

A large flap of skin and temporal muscle was turned down, and the trephine applied an inch and a quarter behind the external frontal eminence, and the same distance above the zygoma. Directly the bone was removed a clot presented itself. The opening in the skull was enlarged downwards and backwards, pulsation of the clot soon became visible, and arterial blood oozed out. After rapidly removing some bone, to the extent altogether of two or three inches square, the bleeding point was found in the posterior branch of the middle meningeal, which had been torn across by a fracture in the bone over it.

I passed a silk ligature on a curved needle through the dura mater, under the artery, and tied the vessel. Bleeding at once stopped. A large quantity of clot was then evacuated. This clot had burrowed downwards towards the base of the brain, as well as over the lateral convolutions ; and in places the brain was separated from the skull for a space of three-quarters of an inch, showing how considerable the pressure must have been.

During the operation the right pupil contracted to the same size as the left, the pulse rose to 160; all signs of compression ceased ; but even on the following day the patient, although quite able to move his left arm, preferred to move his right, and there was some temporary ptosis of right eye-lid.

On June 17th his temperature rose to 100° F., but after this his recovery was rapid and uninterrupted. Before the boy returned to his home a gutta-percha shield was made to protect the large area of brain which was deprived of its bony covering.

It might be wise in these cases to turn down a piece of bone (osteoplastic) instead of using the trephine and bone forceps ; but it is impossible to be certain of the position of the bleeding point. Most of these injuries of the middle meningeal artery are of the anterior branch, it is true, but there was no clinical sign to show that it was the posterior branch that was ruptured in this case, and it would have to be a very large flap of bone to afford access to all possible points of hæmorrhage.

Death is by no means uncommon after these operations, probably from want of expansion of the brain substance, or from œdema following the sudden relief of pressure.

